# The Fontan Operation is Not the End of the Road

**DOI:** 10.5935/abc.20160017

**Published:** 2016-02

**Authors:** Luiz Fernando Caneo, Rodolfo A. Neirotti, Aida Luiza Ribeiro Turquetto, Marcelo Biscegli Jatene

**Affiliations:** 1 Unidade de Cirurgia Cardíaca Pediátrica do Instituto do Coração do Hospital das Clínicas da FMUSP - InCor - HCFMUSP - São Paulo, SP - Brazil; 2 Clinical Professor of Surgery and Pediatrics, Emeritus - Michigan State University - USA

**Keywords:** Heart Defects, Congenital/physiopathology, Heart Defects, Congenital/surgery, Fontan Procedure/trends, Ventricular Function

The purpose of this essay is to increase the awareness on what patients, families and those involved in the treatment and follow-up can face in the long run regarding the survivors of the Fontan operation (FO) and to provide some clues to diminish the deleterious effects of the single ventricle (SV) physiology. Although a significant number of patients survive and are initially asymptomatic, most probably due to adaptation to their limitations, an active approach without being pessimistic is needed in order to prevent problems and thereby improve the long-term prognosis of these patients.

When William Harvey described the circulation he stated: "*Those who believe that one ventricle can drive blood full of spirit into the body and the lungs likewise, are heretics. They forget that nature, being divine, never gave a heart to any where there was no need.*" When Fontan and Baudet published their procedure they advised us that "*this procedure is not an anatomical correction, which would require the creation of a right ventricle, but a way of physiological pulmonary blood flow restoration, with suppression of right and left blood flow mixing*".^1^ They also described the "Ten commandments", a list of precise recommendations regarding the indications for surgery.^2^ Some of the problems that we see today are the result of not following their guidelines. Although they clearly defined the main purpose of this procedure, they could not anticipate the long-term implications of this improvement.

More than forty years later, we are witnessing the consequences of not having a sub-pulmonary ventricle. Although clinical experience shows that to survive we do not need a pumping chamber to drive blood into the lungs, the non-pulsatile flow of both vena cava directly connected to the pulmonary circulation results in the following unintended consequences: 1) *elevated central venous pressure*, and 2) *suboptimal cardiac output*, relative to the normal two-ventricle state due to: a) impaired chronotropic response to exercise; b) decreased capability to transport a normal blood volume across the pulmonary vascular bed, resulting in reduced ventricular filling and low stroke volume; c) inability to adequately increase stroke volume during periods of increased demand; and, d) elevated systemic vascular resistance with deprived tissue perfusion and anaerobic metabolism.

## Are we changing the natural history of the SV physiology?

Presently, this operation is the gold standard destination for patients with SV physiology. Along the years, techniques and strategies have evolved from the original right atrium to pulmonary artery connection to the lateral tunnel and the extra-cardiac conduit, including staged procedures and fenestrations. Better results with this operation have been assigned to these technical modifications. There are many data showing excellent early and long-term outcome with good survival rate.^3-8^ However, despite the remarkable improvements in quality of life and prognosis of patients treated by the FO, there is a decreased exercise capacity and a suboptimal ventricular performance, subsequent to the reduced preload of the functional SV. In addition, these patients often develop scoliosis, kyphosis, have small lungs and consequently a restrictive pulmonary pattern due to previous thoracic surgical procedures. Altogether, it affects different subsystems with a negative impact on functional status, quality of life and the long-term transplant-free survival ratio. Multiple studies assessing the results of the FO demonstrated a decrease in survival with a continuous attrition 15 years after the procedure, regardless of the surgical type of cavopulmonary connection.^9^ In a recent single-institutional study, the actuarial freedom from death or transplantation was 87%, 83%, and 70% at 15, 20, and 25 years respectively after surgery. In this group, death was sudden and unexplained in 9%, thromboembolic in 8%, and heart failure-related in 7%.^10^ In another study assessing morphologically left SV, actuarial survival was 73% at 15 years. Atrial arrhythmias were present in 57%, protein-losing enteropathy in 9%, and thromboembolic events in 6%. In other words, odds are 1 out of 4 that a child after FO will be dead by the time he or she reaches their late 20s.^11^ In a large multi-center cohort, the Pediatric Heart Network (PHN) reviewed 546 children that were on average 11.9 years of age at the time of the study and 8.5 years after the FO. Stroke or thromboembolism was seen in 8% of the patients, exercise performance was abnormal and peak oxygen consumption was only 65% of the predicted for age and gender in a relatively young group.^12^ Adolescents ("older" patients) fared worse than the younger children, suggesting a time-related decrement in functionality.^13^ In another PHN study, parent-reported patient morbidities included deficits in vision in 33%, speech in 27%, and hearing in 7%, as well as problems with attention in 46%, learning in 43%, development in 24%, behavior in 23%, anxiety in 17%, and depression in 8%.^14^

In our center, Turquetto et al.^15^ recently reported suboptimal cardiac function, diminished lung volumes and capacities, as well as reduced respiratory muscle strength in asymptomatic patients - the so-called "perfect Fontan".^15,16^ These are important components of a complex system in which performance and outcomes depend on intricate dynamic interactions that could explain the deficiencies found in the late postoperative period. In other words, despite the low early mortality, when we assess long-term morbidities, the number of patients that are free from problems is low. These findings cannot be ignored, and certainly do not reflect a successful management strategy for patients with single-ventricle physiology.

In summary, despite the good early results, long-term survivors could experience some of the following complications:

- Arrhythmias
- Thromboembolism
- Delayed somatic growth
- Deferred pubertal development
- Protein losing enteropathy (PLE)
- Plastic bronchitis (PB)
- Exercise intolerance
- Liver fibrosis
- Renal dysfunction
- Venous insufficiency

The above mentioned issues justify a regular and careful follow-up of these patients every three to four-year intervals, with major testing at 10 years after FO. The health status of children and adolescents after FO is suboptimal and the management of the late complications represents a significant challenge. If possible, every patient should have serial echocardiogram studies, cardiopulmonary test, abdominal ultrasound, whole body dual energy X-ray absorbtiometry scan and complete blood analysis including CBC with differential, electrolytes, liver enzymes, GGT, total protein, albumin, parathyroid hormone, vitamin D 25-hydroxy and serum ionized Ca^+2^. Urine calcium/creatinine for nutritional assessment, B-natriuretic peptide, cystatin C and basic immune panel should also be performed.^17^

In the late follow-up, we might face a failing Fontan. Failing Fontan patients have two modes of presentation: a) impaired ventricular function and b) those with preserved ventricular function, but with failing subsystems such as PLE and PB. As many as two thirds of adult Fontan patients who die or require transplantation do so with preserved ventricular function.^18^

Due to the increasing amount of patients that are being palliated with FO, the number of children, adolescents, and young adults requiring late rescue therapy with heart transplantation will increase.^19^ The insufficient availability of donors and the associated morbidities with immunosuppression make it imperative that this scarce resource is used appropriately by optimizing the timing of transplantation. Therefore, it is important to identify high-risk patients with failing Fontan physiology and diastolic dysfunction that might benefit from other modes of therapy. Ventricular assist devices (VAD) that may bridge patients more successfully are needed to better prepare the SV circulation over months in order to improve transplant outcomes through better patient selection.^20^

Children and adults with previous procedures undergoing heart transplantation require more complicated operations that should be performed by skilled surgeons. Outcomes of heart transplantation in children with Congenital Heart Disease (CHD) have repeatedly been shown to be inferior to those in children with cardiomyopathy.^21^

Although several centers have described that heart transplantation after the FO is associated with poorer outcomes when compared with those due to other forms of CHD, recent report has shown outstanding outcomes in the former group, comparable to those in children receiving transplants for cardiomyopathy.^22^ Several factors have contributed to the substandard results in the Fontan group, including allosensitization, pulmonary hypertension, challenging operation because of multiple prior sternotomies, complex venous anatomy, requirement of concomitant pulmonary artery reconstruction and the presence of collaterals with subsequent risk of bleeding. In addition, poor clinical condition due to protein-losing enteropathy, malnutrition, liver and kidney dysfunction are aggravated conditions.^22^ We speculate that early referral in better clinical conditions could be responsible for the outstanding results described in this recent publication.

## Where are we going?

Considering the late results of SV palliation, we should strongly consider following the right indications and timing for each step, not based on symptoms, but programmed in advance. Following the internationally recognized algorithm, early in life we ought to band the pulmonary artery to protect the pulmonary vasculature and prevent high pulmonary vascular resistance in those with high pulmonary blood flow, or to create a shunt in those with hypoxemia. The next step should be to diminish the SV volume overload by connecting the superior vena cava to the pulmonary artery - bidirectional Glenn operation - at 3 to 6 months and completing the FO at 2 to 4 years of age.

At the end, we might face two different undesirable scenarios:

- Pump failure that will need heart transplantation at the right time and before clinical deterioration.
- Those with the negative impact of the SV physiology on other subsystems, due to the lack of sub-pulmonary ventricle, which can benefit from a VAD to gain time to optimize patient's condition for transplantation.^23^ The latter has a greater risk of death when compared with the group that presents with poor ventricular function.

Further studies need to be performed to better understand the "unnatural" state of Fontan patients. Strategies targeted toward improving cardiac output and reducing central venous pressure can improve their overall well-being and mitigate the deleterious impact of the new physiology. Further research on methods to improve the circulation through additional interventions - pharmacological, mechanical and even exercise training - is required.
